# Supplementary material for: A Human Health Risk Assessment of Trace Elements Present in Chinese Wine
Source: Molecules. 2019 Jan 11;24(2):248. doi: 10.3390/molecules24020248 (PMC6359022; doi:10.3390/molecules24020248)
Supplement: Supplementary file 1 [file molecules-24-00248-s001.pdf]

Supplementary figure

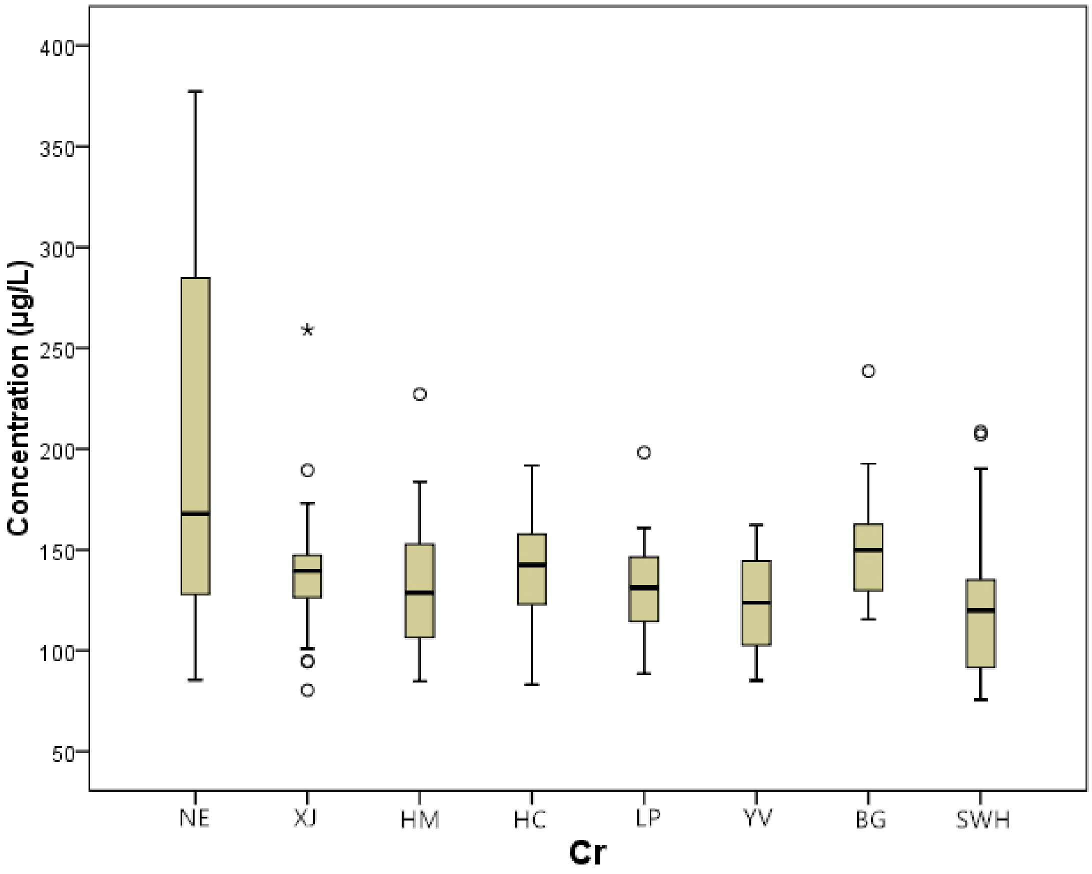

Figure S1. Concentration range and distribution of chromium (Cr) in wines from eight different regions (HM: Helan Mountain; XJ: Xinjiang; HC: Hexi Corridor; BG: Bohai Gulf; SWH: Southwest Highland; YV: Yanhuai Valley; NE: Northeast; LP: Loess Plateau).

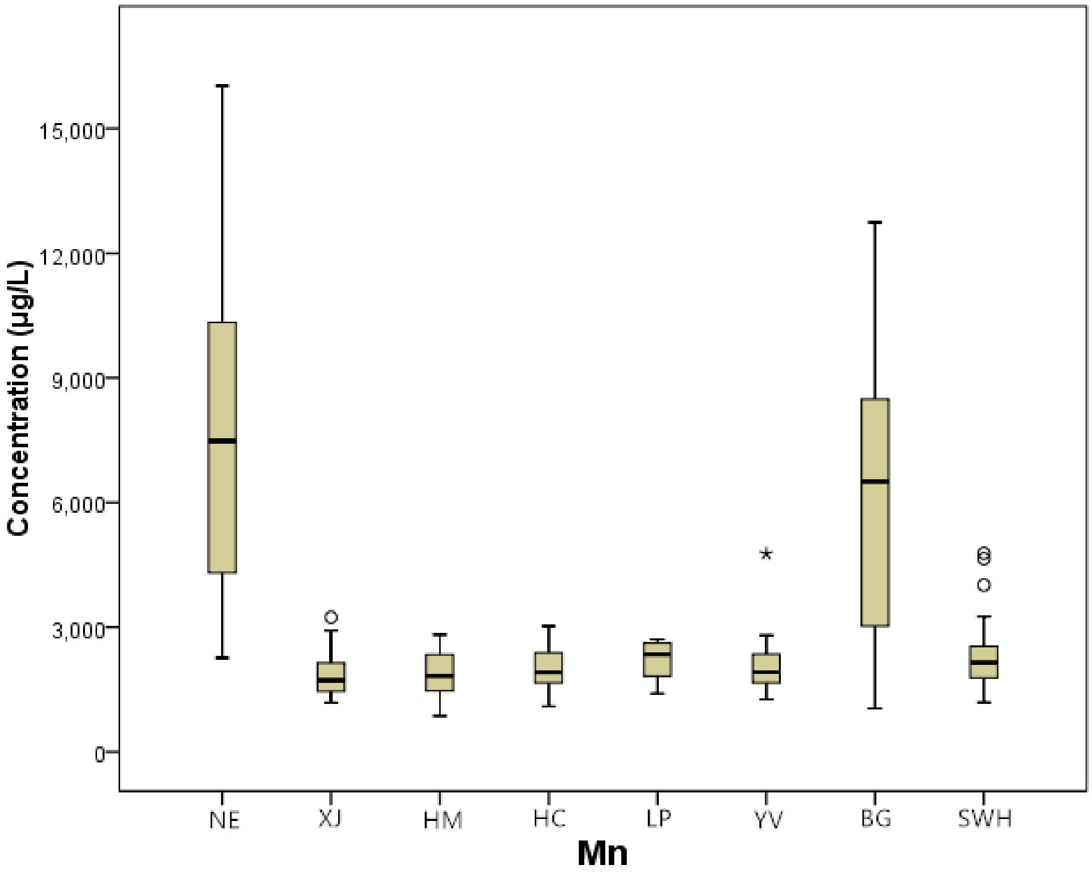

Figure S2. Concentration range and distribution of manganese (Mn) in wines from eight different regions (HM: Helan Mountain; XJ: Xinjiang; HC: Hexi Corridor; BG: Bohai Gulf; SWH: Southwest Highland; YV: Yanhuai Valley; NE: Northeast; LP: Loess Plateau).

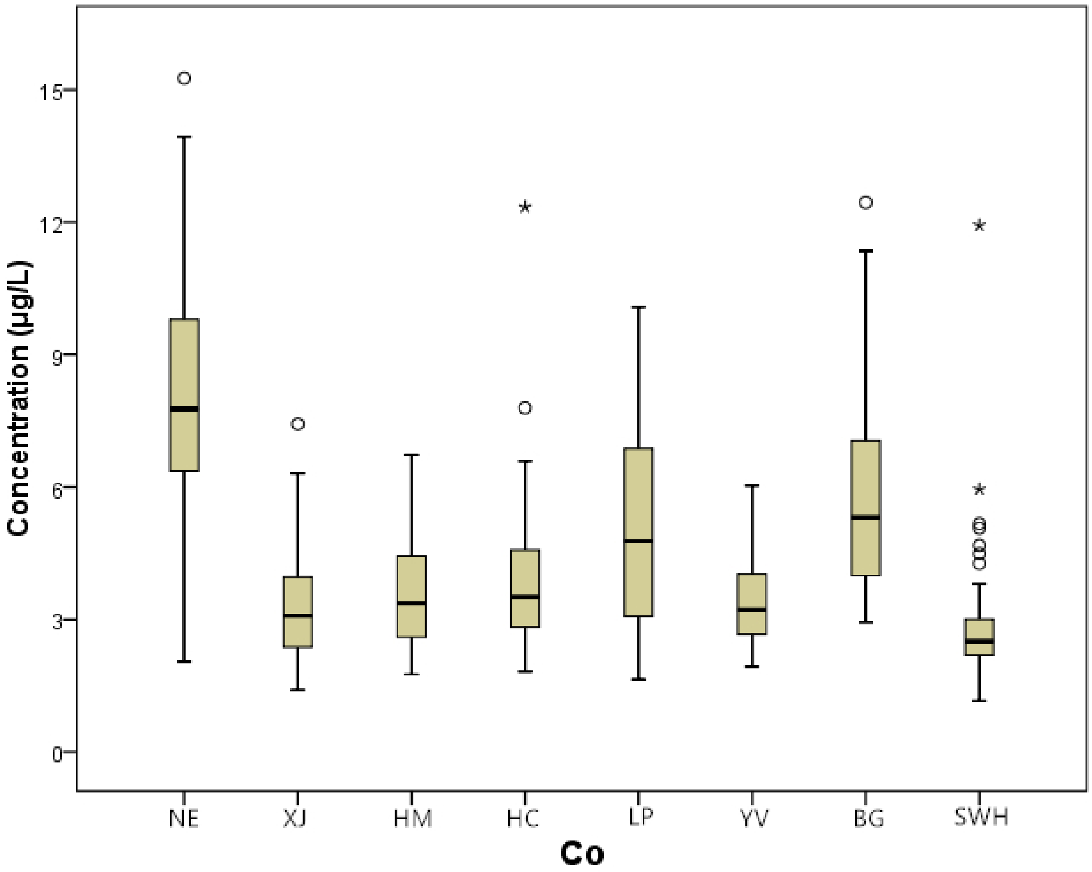

Figure S3. Concentration range and distribution of cobalt (Co) in wines from eight different regions (HM: Helan Mountain; XJ: Xinjiang; HC: Hexi Corridor; BG: Bohai Gulf; SWH: Southwest Highland; YV: Yanhuai Valley; NE: Northeast; LP: Loess Plateau).

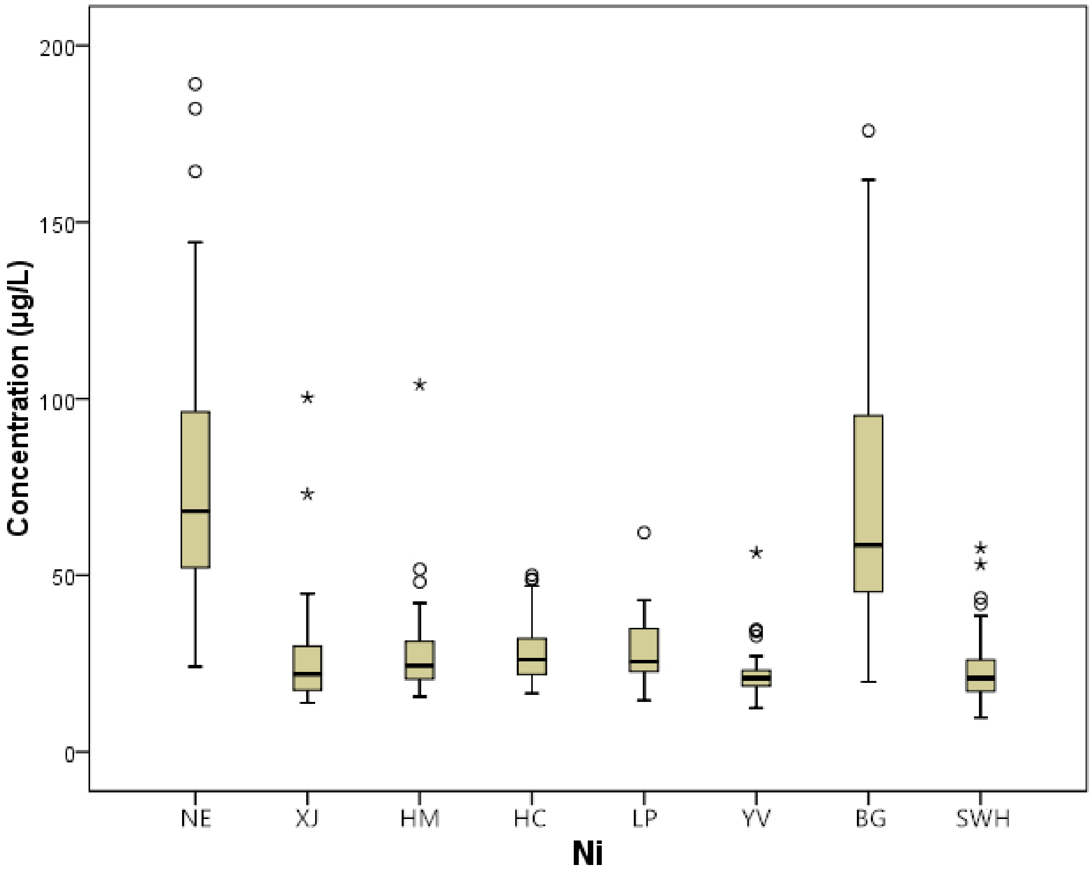

Figure S4. Concentration range and distribution of nickel (Ni) in wines from eight different regions (HM: Helan Mountain; XJ: Xinjiang; HC: Hexi Corridor; BG: Bohai Gulf; SWH: Southwest Highland; YV: Yanhuai Valley; NE: Northeast; LP: Loess Plateau).

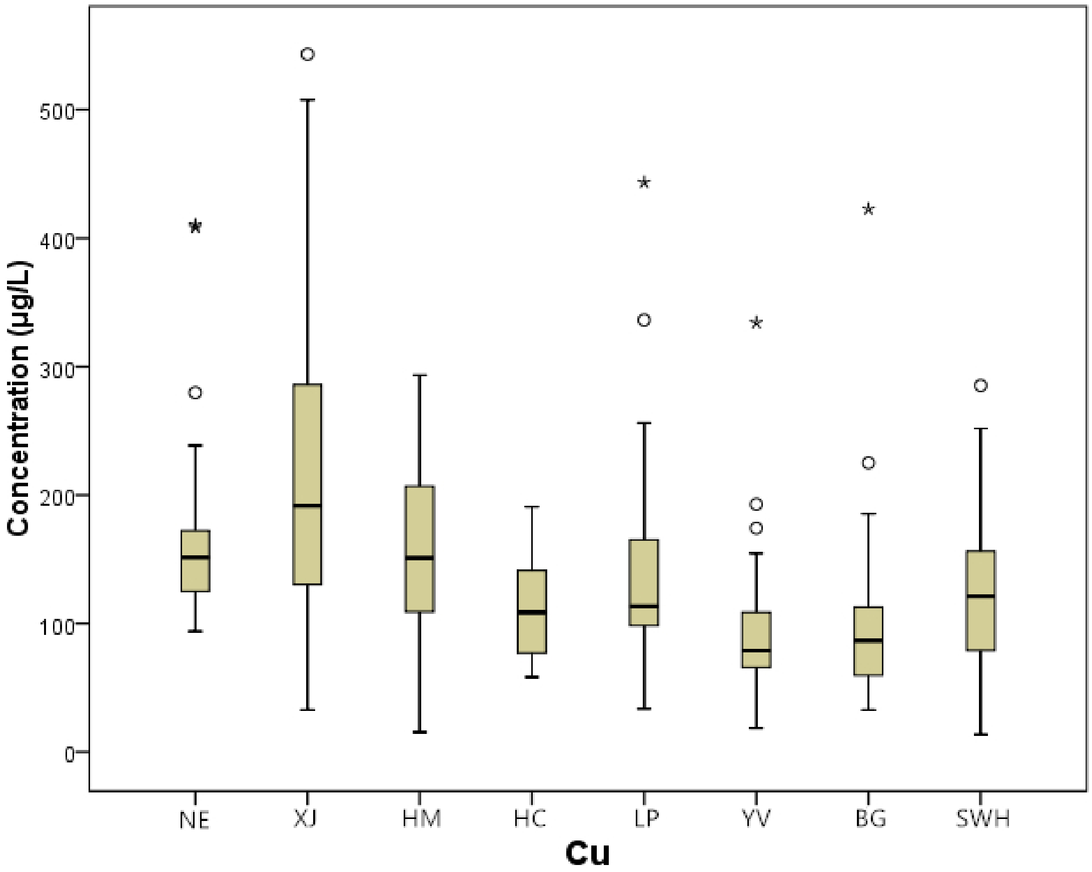

Figure S5. Concentration range and distribution of copper (Cu) in wines from eight different regions (HM: Helan Mountain; XJ: Xinjiang; HC: Hexi Corridor; BG: Bohai Gulf; SWH: Southwest Highland; YV: Yanhuai Valley; NE: Northeast; LP: Loess Plateau).

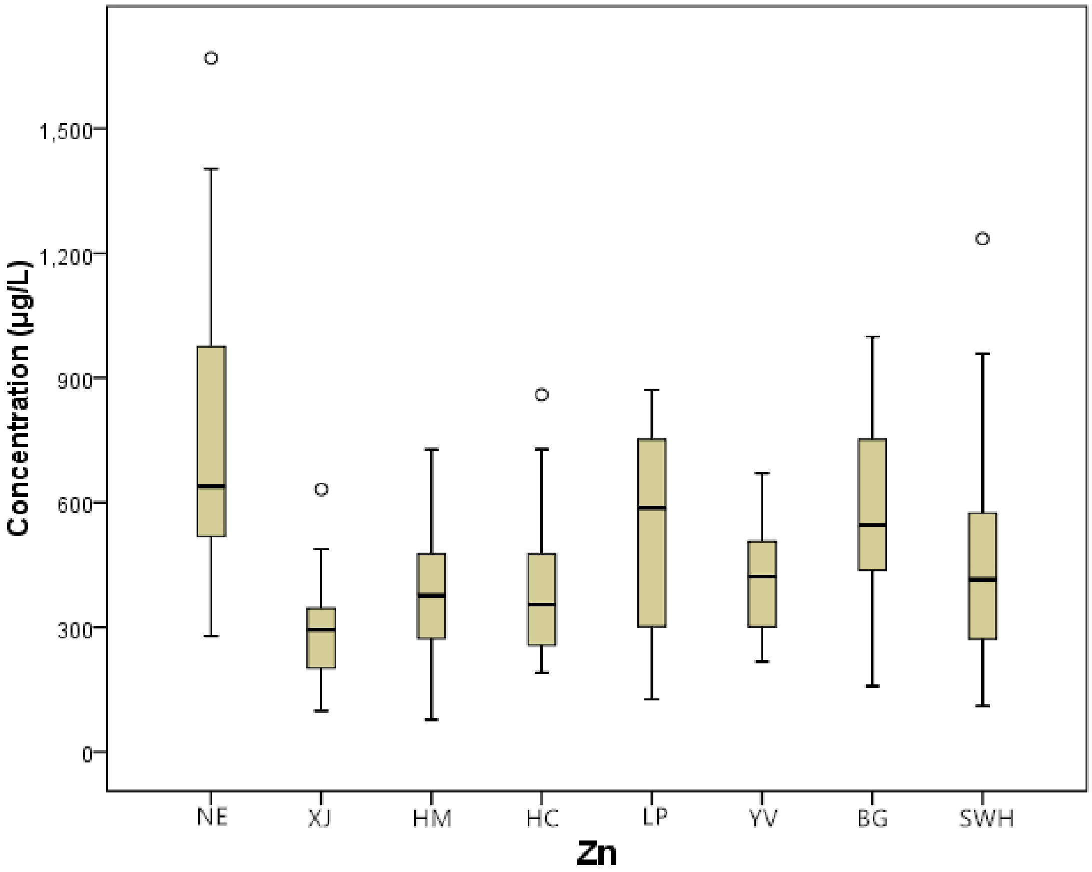

Figure S6. Concentration range and distribution of zinc (Zn) in wines from eight different regions (HM: Helan Mountain; XJ: Xinjiang; HC: Hexi Corridor; BG: Bohai Gulf; SWH: Southwest Highland; YV: Yanhuai Valley; NE: Northeast; LP: Loess Plateau).

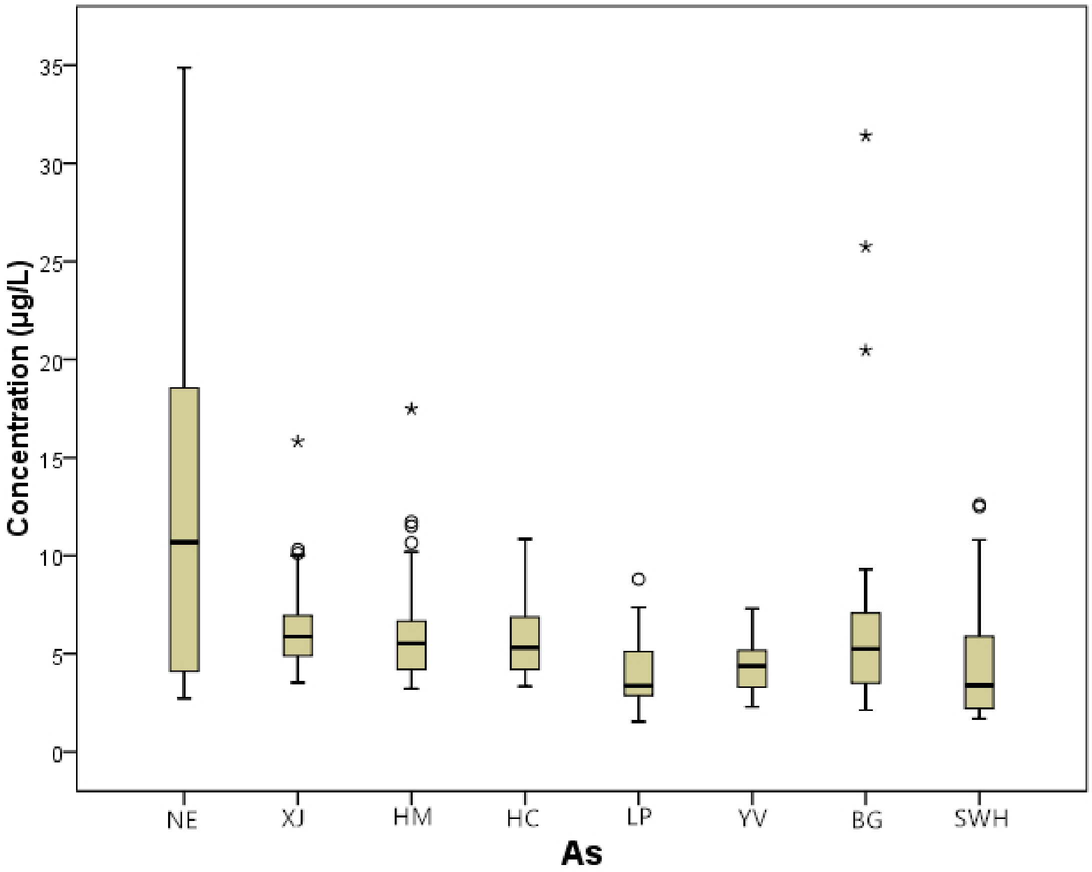

Figure S7. Concentration range and distribution of arsenic (As) in wines from eight different regions (HM: Helan Mountain; XJ: Xinjiang; HC: Hexi Corridor; BG: Bohai Gulf; SWH: Southwest Highland; YV: Yanhuai Valley; NE: Northeast; LP: Loess Plateau).

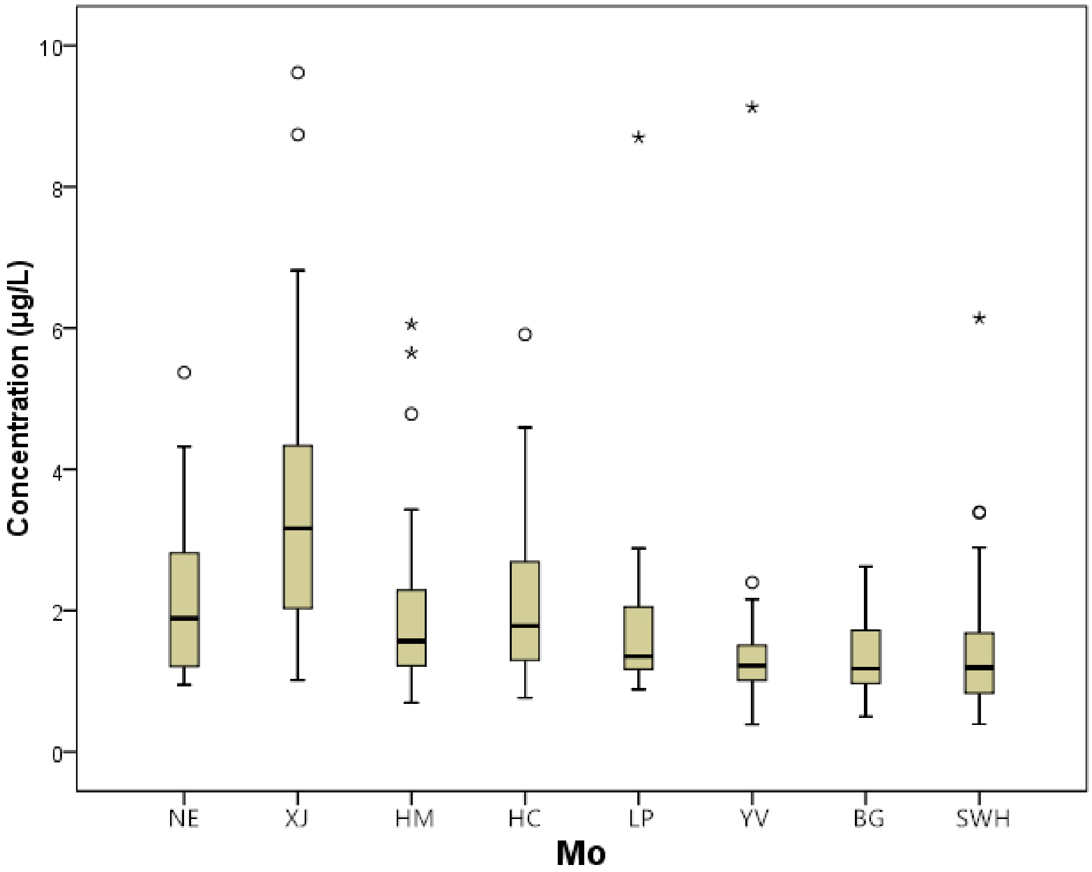

Figure S8. Concentration range and distribution of molybdenum (Mo) in wines from eight different regions (HM: Helan Mountain; XJ: Xinjiang; HC: Hexi Corridor; BG: Bohai Gulf; SWH: Southwest Highland; YV: Yanhuai Valley; NE: Northeast; LP: Loess Plateau).

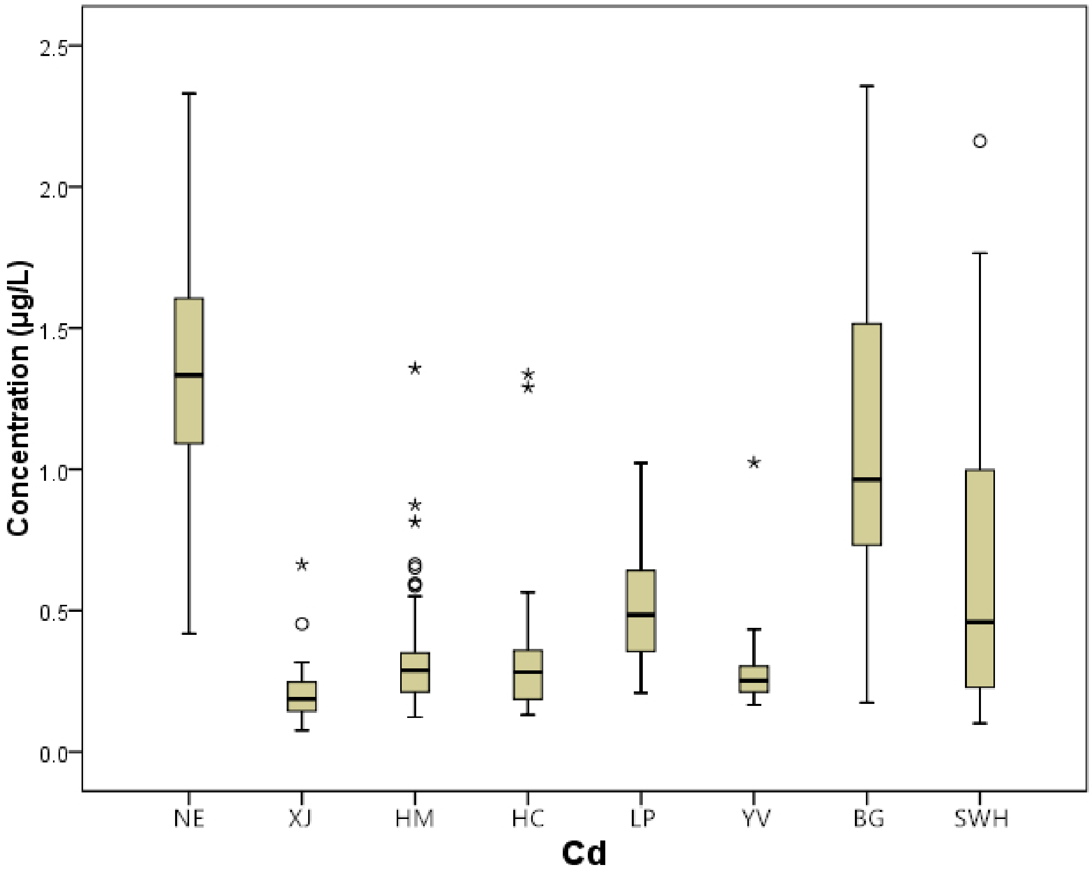

Figure S9. Concentration range and distribution of cadmium (Cd) in wines from eight different regions (HM: Helan Mountain; XJ: Xinjiang; HC: Hexi Corridor; BG: Bohai Gulf; SWH: Southwest Highland; YV: Yanhuai Valley; NE: Northeast; LP: Loess Plateau).

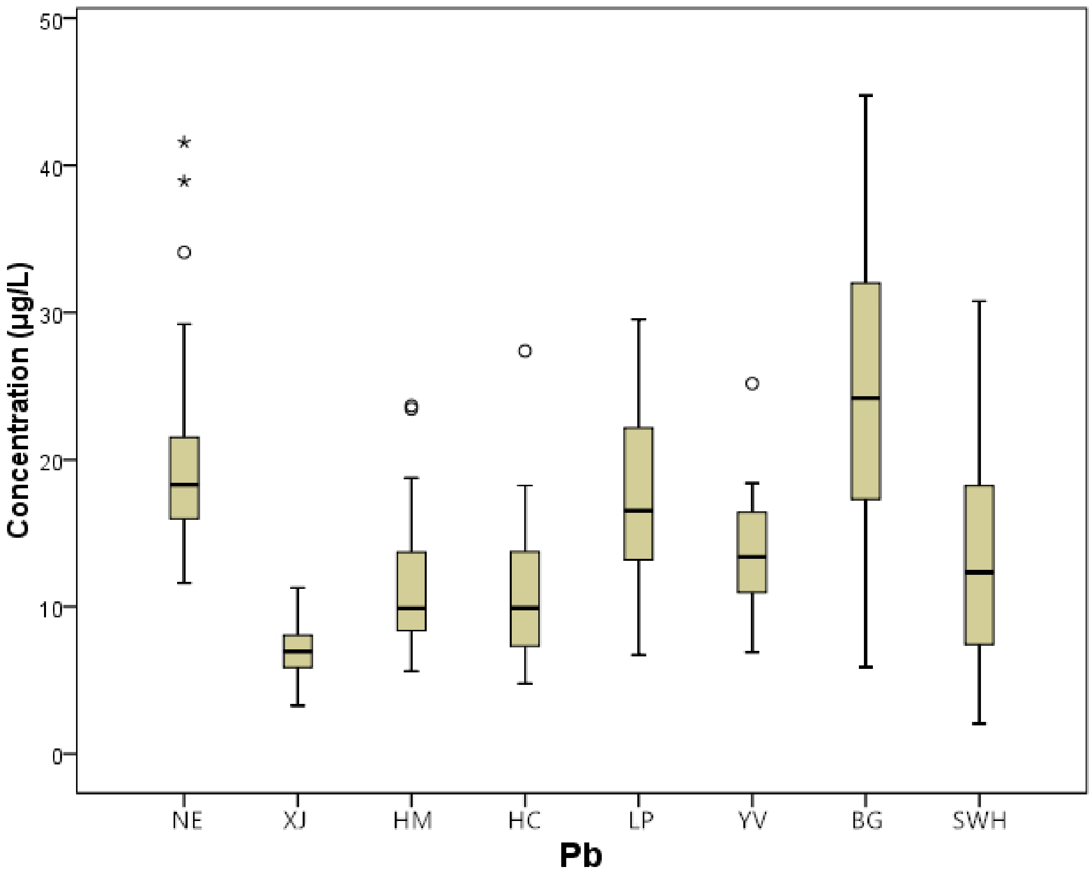

Figure S10. Concentration range and distribution of lead (Pb) in wines from eight different regions (HM: Helan Mountain; XJ: Xinjiang; HC: Hexi Corridor; BG: Bohai Gulf; SWH: Southwest Highland; YV: Yanhuai Valley; NE: Northeast; LP: Loess Plateau).

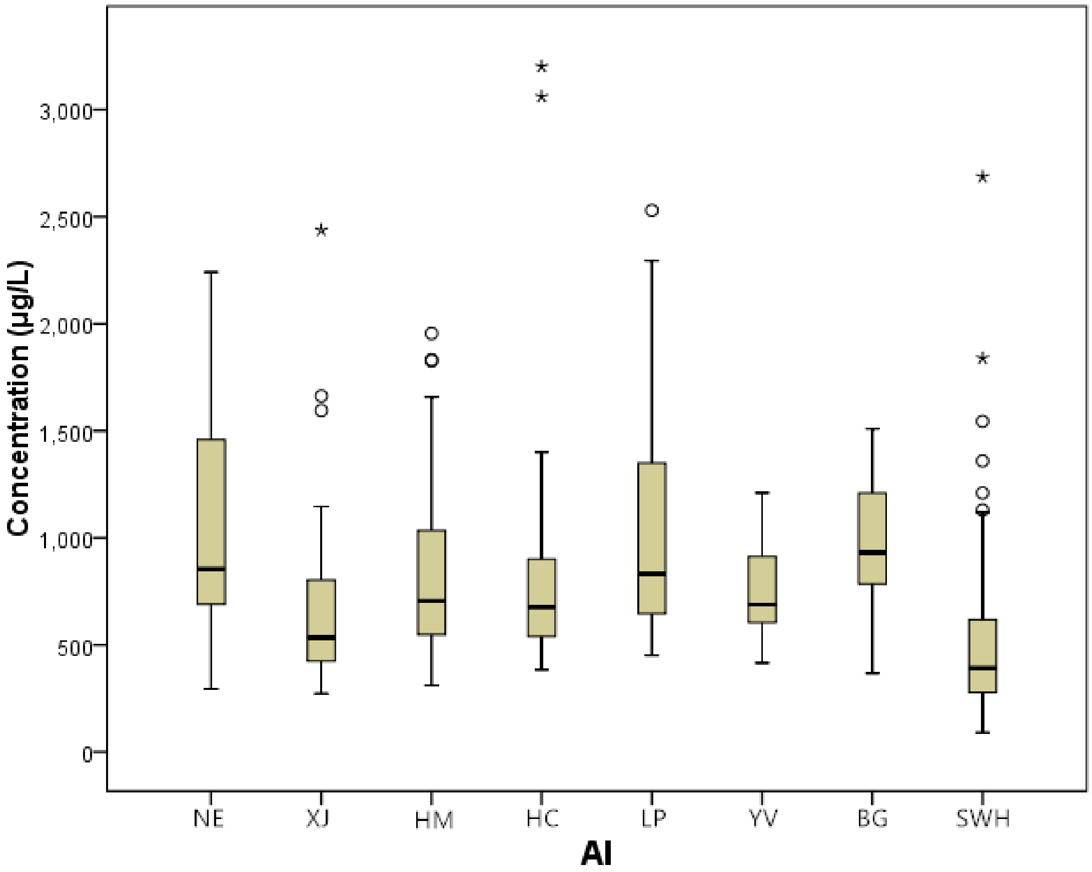

Figure S11. Concentration range and distribution of aluminum (Al) in wines from eight different regions (HM: Helan Mountain; XJ: Xinjiang; HC: Hexi Corridor; BG: Bohai Gulf; SWH: Southwest Highland; YV: Yanhuai Valley; NE: Northeast; LP: Loess Plateau).

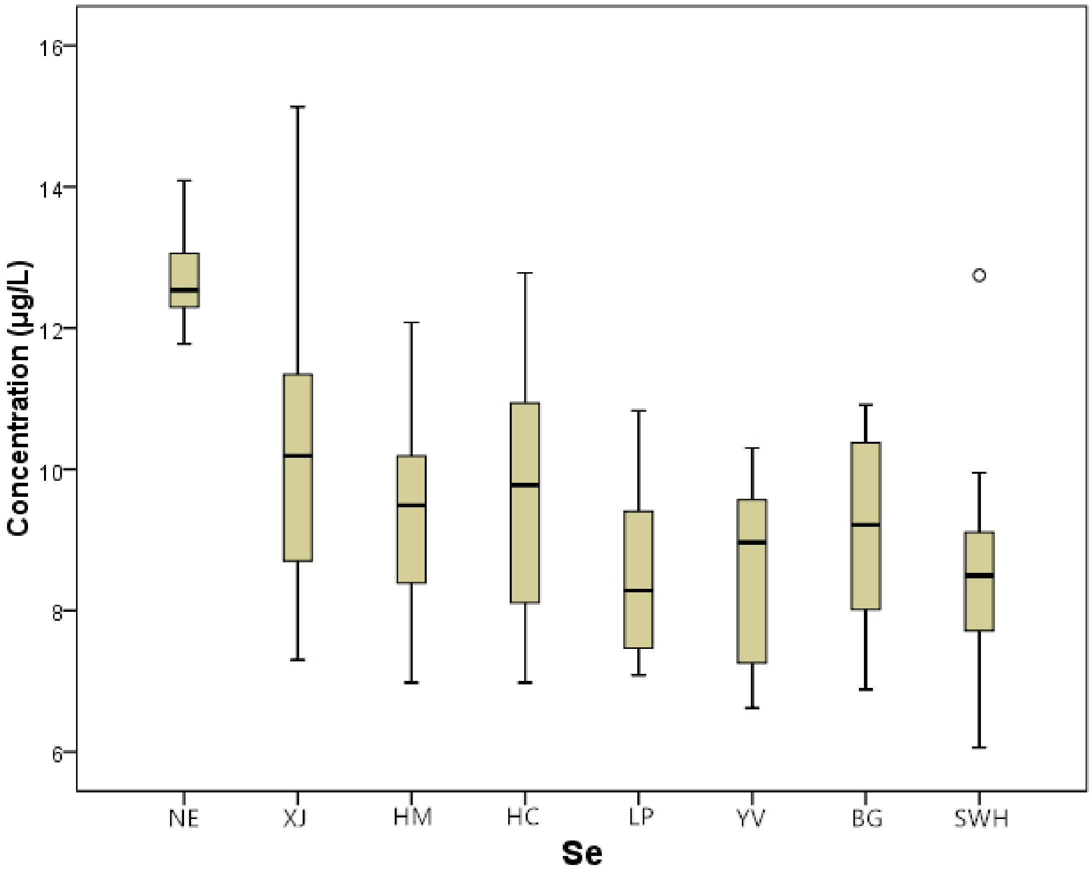

Figure S12. Concentration range and distribution of selenium (Se) in wines from eight different regions (HM: Helan Mountain; XJ: Xinjiang; HC: Hexi Corridor; BG: Bohai Gulf; SWH: Southwest Highland; YV: Yanhuai Valley; NE: Northeast; LP: Loess Plateau).

Supplementary table

Table S1. Determination results of trace elements in 315 wines (μg/L).

Wine	Cr	Mn	Co	Ni	Cu	Zn	As	Mo	Cd	Pb	Al	Se
NE01	128.0	5153	4.98	76.70	99.5	321.2	23.85	2.96	1.177	14.58	1106.5	12.48
NE02	160.6	3881	9.83	61.59	180.8	518.8	14.46	4.17	1.075	34.08	1417.3	13.59
NE03	85.4	3923	2.05	24.11	93.7	975.3	2.73	0.95	0.854	11.61	294.5	11.78
NE04	118.4	3063	5.91	38.17	117.8	279.1	7.53	1.56	1.033	21.30	1468.0	12.01
NE05	150.3	4242	9.85	53.48	157.1	497.1	15.32	4.27	1.047	35.91	3994.8	13.44
NE06	229.7	3610	4.41	76.25	151.6	594.7	4.10	1.17	1.134	13.43	689.4	12.32
NE07	369.3	10602	10.31	74.40	279.8	1403.6	18.96	1.88	1.605	29.21	2152.3	13.85
NE08	149.9	8535	10.16	57.18	207.5	635.8	5.70	2.04	1.429	21.51	2116.5	12.59
NE09	284.8	4312	6.51	54.91	168.3	1378.9	11.39	5.37	1.447	25.57	493.3	12.73
NE10	313.6	6483	8.03	52.22	410.4	741.5	34.88	3.09	1.025	16.82	1458.7	12.94
NE11	99.0	6758	7.60	47.38	143.8	383.8	4.57	1.21	1.190	20.59	1249.0	12.39
NE12	327.3	6469	7.05	70.60	151.2	1669.6	9.99	2.31	1.426	18.01	719.6	12.96
NE13	316.6	6560	9.33	103.62	2108.6	925.0	68.46	3.50	1.214	26.56	1268.6	13.80
NE14	285.0	4763	6.74	100.96	299.8	1147.9	41.57	4.24	1.199	22.81	860.2	13.19
NE15	356.8	9724	9.54	69.56	130.1	2124.8	11.01	1.47	1.784	54.51	723.8	13.51
NE16	139.0	9523	11.78	65.74	162.1	642.8	19.36	1.16	1.435	16.67	852.5	12.99
NE17	168.3	11464	13.93	96.28	169.1	647.9	11.41	1.33	1.688	17.81	950.0	12.30
NE18	112.3	10338	6.81	51.00	152.7	661.6	30.19	1.14	1.408	14.89	437.4	12.87
NE19	136.5	15670	15.26	62.04	238.6	669.9	21.92	1.38	1.946	15.96	719.2	12.44
NE20	246.4	9633	7.46	236.68	97.0	746.4	3.77	2.57	0.989	20.71	2304.0	12.86
NE21	159.1	5525	7.17	116.20	408.4	499.7	14.84	1.13	1.034	18.32	677.2	12.20

Wine	Cr	Mn	Co	Ni	Cu	Zn	As	Mo	Cd	Pb	Al	Se
NE22	247.4	16026	10.46	182.15	160.4	477.9	3.28	2.30	1.111	17.91	856.8	12.30
NE23	260.5	2264	5.89	164.37	98.2	347.5	4.08	3.28	0.586	18.26	2241.1	12.71
NE24	137.6	30466	19.99	79.18	115.5	836.7	6.68	1.86	3.468	45.15	3209.8	12.02
NE25	167.4	3521	7.92	56.40	102.1	335.6	5.40	1.30	1.735	21.53	1500.5	12.20
NE26	261.7	7785	6.90	53.12	101.8	1006.0	30.46	2.82	2.331	26.38	1185.2	13.92
NE27	345.2	15010	9.80	79.05	150.0	1248.1	12.88	3.17	2.040	19.72	846.1	13.13
NE28	112.5	7186	6.50	38.92	164.4	595.5	18.56	1.07	1.376	11.87	458.2	12.34
NE29	377.3	11790	9.42	144.30	149.1	697.6	5.47	1.29	1.091	19.99	691.2	13.06
NE30	146.8	5915	6.36	103.79	125.0	529.7	3.53	1.90	1.094	16.59	500.7	12.21
XJ01	139.6	2278	4.57	20.16	224.9	286.3	5.84	3.37	0.271	6.02	1146.1	9.10
XJ02	80.4	2322	3.10	16.11	351.3	133.2	5.03	3.36	0.144	5.32	726.7	7.30*
XJ03	259.0	1814	3.76	100.31	142.5	384.4	5.25	3.97	0.273	8.27	692.9	10.62
XJ04	147.2	1739	2.52	29.75	120.4	412.2	4.25	2.17	0.233	7.42	768.8	8.20
XJ05	155.6	1989	2.80	15.13	197.3	250.1	6.40	3.00	0.277	7.92	451.7	12.25
XJ06	143.4	1775	3.30	19.70	242.4	249.5	5.75	2.34	0.170	7.94	473.4	11.61
XJ07	101.5	2187	4.06	33.00	96.3	244.1	5.26	4.34	0.303	5.40	598.2	8.40
XJ08	136.6	1218	2.93	13.93	152.2	294.2	7.73	5.61	0.171	6.31	507.7	12.52
XJ09	116.6	1614	3.39	17.92	192.9	334.7	8.22	5.96	0.228	7.31	586.2	15.13
XJ10	137.6	1360	5.57	28.82	216.1	342.8	4.08	5.46	0.168	5.46	307.3	9.39
XJ11	138.7	1475	2.48	24.30	301.4	125.8	4.96	5.48	0.221	6.15	339.0	12.38
XJ12	106.8	1707	2.93	18.10	361.9	274.4	7.35	2.49	0.185	7.08	338.1	11.29
XJ13	139.9	1196	2.34	14.32	415.7	220.0	6.01	2.39	0.087*	3.68	275.9	8.58
XJ14	189.5	2617	6.31	73.00	163.7	302.2	5.90	4.16	0.453	6.41	867.7	8.76
XJ15	163.1	1397	2.84	13.83	507.9	333.6	6.51	1.01	0.162	4.05	596.9	8.67
XJ16	141.3	1534	1.51	14.92	194.4	160.8	3.91	1.80	0.133	5.77	448.2	10.19

Wine	Cr	Mn	Co	Ni	Cu	Zn	As	Mo	Cd	Pb	Al	Se
XJ17	146.9	1612	2.68	23.92	449.2	109.8	5.72	3.36	0.088*	5.10	819.5	12.77
XJ18	145.6	1293	2.23	15.87	345.1	174.2	5.82	4.55	0.156	6.01	533.3	11.34
XJ19	136.5	1388	2.25	26.50	285.9	134.2	7.90	4.83	0.129	8.06	507.3	9.30
XJ20	139.3	1183	1.40	16.91	130.2	98.8	3.88	2.46	0.076*	8.27	608.6	7.37*
XJ21	152.2	1638	2.37	44.82	47.7	344.9	4.07	1.52	0.146	4.02	316.1	8.07
XJ22	132.7	1465	2.34	19.70	141.8	240.3	3.76	1.42	0.145	5.88	510.4	7.78*
XJ23	126.9	1353	2.27	21.55	142.8	245.2	3.53	1.94	0.103*	6.45	534.8	7.83*
XJ24	125.8	1231	2.31	23.99	169.4	176.5	3.88	2.75	0.139	13.56	557.7	8.53
XJ25	147.6	1947	4.05	23.28	278.6	420.3	4.91	4.33	0.253	8.83	897.1	10.59
XJ26	150.9	1667	3.36	27.93	543.3	144.6	6.10	3.49	0.138	7.23	1032.5	13.45
XJ27	149.1	2031	4.01	35.43	37.9	336.2	9.04	4.46	0.299	11.28	815.9	9.97
XJ28	127.7	1757	4.81	36.70	32.6	409.4	15.82	4.16	0.247	9.01	715.0	10.26
XJ29	120.5	1574	3.26	18.22	157.4	381.0	5.29	2.71	0.155	4.44	356.6	9.11
XJ30	112.2	1581	3.07	43.79	125.0	304.1	5.25	2.03	0.193	7.05	272.0	11.40
XJ31	121.4	1637	1.68	17.37	385.3	100.6	6.58	4.60	0.099*	7.76	288.2	8.40
XJ32	151.9	1445	3.69	15.13	402.9	154.5	7.96	9.62	0.116*	4.68	512.6	10.26
XJ33	142.6	2488	4.54	34.44	89.3	488.5	4.90	1.33	0.223	9.55	1109.5	10.73
XJ34	126.3	1351	2.02	14.58	82.3	169.6	10.33	3.07	0.160	6.89	338.6	12.25
XJ35	145.5	1818	2.83	34.61	164.0	218.6	8.14	2.34	0.122*	3.81	322.2	12.46
XJ36	173.1	2023	3.19	29.94	169.2	280.2	7.03	2.40	0.169	6.09	424.9	12.16
XJ37	146.9	1457	2.80	32.03	177.1	290.8	4.95	3.51	0.120*	3.28	316.2	10.53
XJ38	106.4	1750	1.85	14.94	190.2	178.7	6.00	3.26	0.244	7.53	503.6	9.97
XJ39	144.6	2596	7.43	30.39	113.8	631.8	6.39	1.71	0.663	10.13	2437.7	7.42*
XJ40	134.0	1431	2.22	17.83	136.9	335.5	3.82	4.15	0.197	8.10	681.8	11.42
XJ41	138.8	1780	2.10	20.43	185.4	201.6	6.74	5.13	0.174	6.63	508.8	9.48

Wine	Cr	Mn	Co	Ni	Cu	Zn	As	Mo	Cd	Pb	Al	Se
XJ42	100.9	1649	2.41	19.92	200.6	317.5	6.95	2.33	0.195	7.22	446.1	10.90
XJ43	142.6	2206	3.23	22.72	260.2	452.2	6.46	1.34	0.309	8.95	649.0	10.50
XJ44	156.3	2916	4.82	26.63	421.8	311.3	6.59	1.48	0.211	9.72	1054.4	10.21
XJ45	157.0	3240	5.25	28.75	427.1	357.4	6.11	1.47	0.189	9.46	1020.3	10.19
XJ46	138.0	2141	3.96	20.84	78.9	312.3	10.11	8.74	0.207	6.64	1595.2	7.59*
XJ47	138.5	2249	4.77	43.12	67.9	293.7	10.01	6.82	0.261	7.15	1663.2	8.70
XJ48	112.5	2394	3.51	20.72	245.6	421.2	4.89	2.89	0.144	5.99	803.5	10.13
XJ49	94.8	2071	2.88	17.46	119.1	398.8	4.33	1.60	0.301	7.13	493.0	10.37
XJ50	94.5	1392	3.38	22.83	225.7	488.5	3.62	1.86	0.243	5.87	329.0	9.62
HM01	159.4	1845	3.45	25.47	276.5	338.4	7.59	3.01	0.595	14.09	587.1	8.86
HM02	108.1	1913	5.68	25.29	242.9	515.6	5.23	2.61	0.280	12.70	1332.7	11.59
HM03	147.5	1548	1.77	15.76	125.7	90.8	6.18	3.17	0.295	9.90	421.6	8.74
HM04	138.4	1309	4.57	37.01	190.7	563.6	10.66	3.00	0.664	23.66	1429.8	9.45
HM05	152.5	1428	1.93	21.48	150.9	219.8	5.46	1.04	0.373	13.95	561.0	8.21
HM06	172.5	1850	4.52	23.49	225.1	553.4	8.96	1.35	0.596	18.76	1037.8	11.38
HM07	124.4	1369	2.31	17.16	98.3	356.6	6.00	0.98	0.652	16.82	533.8	7.63*
HM08	124.6	1495	3.13	21.05	198.2	499.6	10.20	2.13	0.814	14.64	1286.8	7.96*
HM09	157.3	1227	2.48	15.77	15.4	304.5	11.51	3.43	0.289	12.69	803.7	9.61
HM10	113.1	1154	3.38	20.31	197.1	340.3	4.26	6.27	0.188	9.96	741.8	36.54
HM11	124.6	1523	2.98	19.91	109.9	531.0	5.60	1.41	0.337	11.97	499.1	9.79
HM12	183.7	2705	2.66	48.11	40.9	77.2	3.90	3.43	0.123*	5.96	580.1	9.98
HM13	105.0	1239	2.08	15.57	110.0	208.7	3.26	1.74	0.235	11.44	490.5	9.40
HM14	93.8	1567	4.78	32.78	110.0	385.4	11.73	3.42	0.276	12.00	1832.5	10.30
HM15	120.6	1864	6.73	35.53	233.1	529.3	17.48	6.05	0.400	16.41	1826.8	10.35
HM16	154.4	1703	4.34	30.53	62.4	467.6	6.74	1.98	0.539	22.42	1006.3	11.80

Wine	Cr	Mn	Co	Ni	Cu	Zn	As	Mo	Cd	Pb	Al	Se
HM17	148.1	2321	6.94	37.19	87.3	470.4	7.30	2.33	0.409	18.39	1012.0	13.35
HM18	141.8	1783	4.91	28.17	81.2	443.6	8.03	4.78	0.259	16.94	1053.5	11.14
HM19	143.1	1393	3.67	20.40	142.5	358.7	5.80	1.66	0.391	18.22	813.4	8.30
HM20	141.0	1387	4.70	22.24	176.2	230.4	6.17	0.90	0.351	15.04	1059.7	8.19
HM21	102.9	1632	4.28	23.86	194.6	294.1	7.58	5.65	0.226	16.74	1032.5	11.05
HM22	140.9	1448	2.74	31.89	89.1	273.2	5.74	1.92	0.304	13.37	950.7	7.72*
HM23	167.5	1228	2.64	20.84	171.9	250.7	6.92	2.12	0.228	9.88	745.6	11.33
HM24	99.2	1333	2.17	31.59	165.6	224.2	4.58	1.78	0.229	12.22	495.2	9.46
HM25	102.7	1594	2.60	22.25	179.4	251.0	4.59	2.29	0.183	9.76	585.3	10.17
HM26	90.9	1923	3.69	22.49	224.0	368.9	4.41	0.88	0.159	8.25	708.6	9.87
HM27	115.9	2008	3.39	20.62	246.9	445.4	4.69	0.82	0.201	7.72	1049.2	8.00
HM28	84.8	2128	3.32	20.37	161.6	358.3	4.41	1.33	0.210	8.50	618.2	9.51
HM29	116.3	2309	5.72	26.98	132.5	474.4	6.30	1.37	0.222	10.34	1955.3	8.14
HM30	97.4	2368	2.85	21.50	103.3	437.4	3.22	1.69	0.283	7.89	524.9	9.01
HM31	95.7	2266	3.19	20.66	276.9	451.1	3.28	1.62	0.295	8.26	574.0	9.67
HM32	89.1	1932	2.25	15.95	67.3	481.4	3.62	1.17	0.322	8.36	369.7	9.36
HM33	227.2	2431	4.35	104.03	279.6	330.0	3.87	2.01	0.299	7.74	439.8	10.10
HM34	139.0	2187	1.75	22.01	194.7	234.5	4.23	1.34	0.204	8.08	422.4	9.34
HM35	169.3	2820	3.89	36.96	117.8	482.2	4.91	2.24	0.318	7.36	769.0	9.07
HM36	171.8	1945	4.22	25.11	277.0	427.9	7.42	2.36	0.875	10.66	1050.4	9.65
HM37	122.3	1652	2.31	20.65	54.5	383.7	3.24	1.50	0.303	5.63	615.7	7.47*
HM38	112.6	2496	3.53	35.89	128.5	433.2	5.17	2.17	0.311	9.23	650.9	9.34
HM39	121.0	1732	2.12	20.28	139.1	224.2	3.69	1.15	0.186	5.69	725.6	7.23*
HM40	135.3	1804	2.75	28.83	205.7	328.9	3.73	0.94	0.217	9.26	615.2	11.14
HM41	92.6	1070	2.39	36.69	69.4	267.6	4.20	1.40	0.236	7.68	311.5	8.83

Wine	Cr	Mn	Co	Ni	Cu	Zn	As	Mo	Cd	Pb	Al	Se
HM42	103.0	1798	3.38	51.73	64.4	384.1	3.68	1.47	0.341	9.14	486.7	8.69
HM43	112.4	1629	2.82	23.15	269.2	276.5	6.70	1.66	0.394	16.36	607.9	9.85
HM44	89.2	1642	3.11	21.44	173.0	223.8	6.62	1.06	0.350	13.47	701.4	8.87
HM45	130.9	2512	4.73	36.91	74.9	490.5	5.95	1.56	0.217	9.63	1158.8	10.75
HM46	156.0	2530	5.34	42.14	75.1	574.7	6.40	1.23	0.233	8.97	1156.3	8.48
HM47	121.0	2466	3.92	28.34	142.4	407.7	5.11	1.22	0.183	8.39	886.6	10.03
HM48	150.9	2478	4.33	31.04	213.6	273.3	6.26	1.31	0.130	8.43	894.7	9.74
HM49	153.0	2545	4.01	30.21	293.2	302.1	6.23	0.91	0.156	8.03	1006.5	11.54
HM50	95.6	2157	2.69	25.13	185.4	429.1	3.88	0.69	0.161	6.90	602.8	10.20
HM51	95.7	2527	5.71	28.34	143.3	439.4	5.48	1.01	0.192	9.30	1187.8	9.61
HM52	159.6	2659	4.66	28.61	88.0	593.1	6.82	1.49	0.432	13.11	985.0	11.10
HM53	163.0	2385	3.74	19.65	120.3	728.0	5.78	2.84	0.178	8.93	811.8	9.88
HM54	168.2	2536	6.29	31.76	108.1	569.5	8.08	2.29	0.342	12.44	1658.2	10.33
HM55	131.4	2412	5.27	31.97	96.9	577.5	5.58	2.39	0.552	15.88	973.5	10.33
HM56	133.4	2146	5.22	27.10	150.8	478.2	5.10	2.44	0.214	8.69	907.9	8.05
HM57	143.7	2050	3.36	20.29	117.4	454.7	3.83	1.31	0.288	10.48	676.5	9.68
HM58	102.4	1878	3.45	28.96	70.9	413.6	3.13	1.39	0.266	13.48	582.0	10.54
HM59	126.5	863	2.31	18.73	147.0	294.5	3.75	1.32	0.172	7.84	491.2	6.98*
HM60	142.2	1302	2.60	24.69	189.1	213.8	8.37	1.58	0.328	9.44	406.7	7.27*
HC01	168.0	1134	3.20	26.52	147.7	335.4	4.81	2.70	0.177	14.75	646.6	11.14
HC02	154.0	1094	2.76	21.89	100.7	260.0	5.04	2.87	0.141	6.13	638.2	10.94
HC03	99.9	1661	4.57	19.79	61.6	389.7	3.33	3.95	0.153	5.92	588.7	6.98*
HC04	166.1	2385	4.05	25.19	114.7	611.3	6.63	1.54	0.314	13.55	1006.0	9.03
HC05	159.1	2135	4.60	29.29	160.8	427.4	6.95	1.29	0.330	9.33	755.6	10.75
HC06	157.7	1922	4.54	27.34	141.3	604.2	7.33	2.67	0.356	13.75	900.9	11.85

Wine	Cr	Mn	Co	Ni	Cu	Zn	As	Mo	Cd	Pb	Al	Se
HC07	95.6	1641	2.41	16.81	183.9	220.1	3.77	1.72	0.158	18.24	755.5	7.95*
HC08	143.3	1811	4.81	24.69	99.4	694.3	4.12	1.06	1.290	15.02	536.7	7.35*
HC09	142.5	2653	2.74	34.27	77.0	190.5	4.20	1.30	0.234	27.38	744.7	8.73
HC10	122.7	2328	2.50	21.28	165.3	235.1	3.82	1.45	0.209	12.46	451.4	8.68
HC11	83.3	1204	1.82	23.03	146.7	288.3	4.41	2.69	0.186	14.28	474.1	8.76
HC12	118.6	1993	2.27	16.53	130.7	244.8	3.74	1.40	0.156	9.58	383.6	7.93*
HC13	132.0	1942	4.55	22.52	73.5	379.3	6.80	1.85	0.285	5.29	784.0	10.38
HC14	140.3	2371	4.07	25.68	58.1	357.6	8.75	1.59	0.280	9.08	542.9	12.61
HC15	151.1	2614	3.59	24.51	114.2	393.7	6.58	1.54	0.341	9.90	539.1	12.73
HC16	133.8	1975	3.79	20.47	86.7	384.8	4.71	1.35	0.330	6.94	561.3	8.95
HC17	104.6	2531	3.43	19.30	67.3	415.9	4.84	1.33	0.566	10.21	704.6	8.15
HC18	159.5	2438	12.35	47.00	91.9	859.7	10.84	2.75	1.337	17.67	3061.7	9.50
HC19	113.6	1831	3.28	26.94	77.1	343.1	4.52	0.77	0.471	7.33	586.6	8.11
HC20	146.0	1595	4.99	48.91	136.7	349.7	5.50	2.29	0.313	7.67	732.7	10.05
HC21	193.6	3209	16.99	66.31	123.9	923.7	18.41	5.06	0.674	16.34	5163.1	11.28
HC22	128.3	1507	4.10	29.25	136.5	351.2	5.58	2.15	0.278	6.00	844.7	10.50
HC23	191.8	1809	4.09	48.70	74.9	256.6	6.86	4.59	0.358	11.28	1025.8	10.70
HC24	148.3	1679	3.26	27.89	90.5	297.7	7.25	2.41	0.559	8.29	606.7	11.87
HC25	179.1	3030	6.58	31.45	102.9	450.5	9.74	2.03	0.466	9.91	1023.5	10.71
HC26	185.5	2558	7.79	40.77	72.7	728.4	10.77	2.99	0.421	17.61	3202.4	12.78
HC27	165.4	3214	8.71	41.79	182.8	982.4	9.17	4.10	0.491	15.88	3624.6	10.56
HC28	123.1	2428	3.23	23.78	160.4	210.2	3.42	1.25	0.163	10.84	457.8	8.65
HC29	651.2	2122	4.76	381.14	163.4	297.8	3.72	1.68	0.188	12.27	342.9	10.63
HC30	122.9	1803	2.11	17.70	190.9	219.4	3.61	0.94	0.132	8.67	421.7	7.67*
LP01	137.0	2045	3.49	37.69	94.4	428.4	4.66	2.31	1.187	91.06	1701.6	9.27

Wine	Cr	Mn	Co	Ni	Cu	Zn	As	Mo	Cd	Pb	Al	Se
LP02	132.5	2007	4.12	22.08	256.0	775.5	3.38	8.70	0.272	6.71	1019.9	8.54
LP03	107.1	2643	7.83	25.07	132.3	871.7	5.15	1.89	0.490	15.31	1917.7	9.73
LP04	88.4	1621	4.12	21.17	163.9	304.6	2.78	0.97	0.402	12.86	450.3	8.87
LP05	130.1	1400	2.13	14.55	336.3	299.0	2.33	0.88	0.358	8.53	632.1	7.27*
LP06	129.4	1778	1.65	20.06	188.8	126.3	1.54	1.76	0.208	12.62	591.3	7.44*
LP07	97.3	1618	1.96	26.00	128.0	197.0	3.26	1.39	0.391	25.23	582.9	10.06
LP08	159.5	1741	7.82	40.96	33.5	329.5	5.17	2.20	0.672	29.53	2295.2	7.54*
LP09	120.5	2333	5.47	30.97	109.9	585.5	7.37	1.16	0.612	19.35	878.4	9.01
LP10	131.2	2709	4.23	24.51	102.3	727.8	4.24	1.12	0.485	15.42	848.0	7.20*
LP11	146.3	2225	5.36	29.23	91.1	269.9	3.36	1.18	0.572	21.59	1498.4	7.14*
LP12	103.2	2606	3.96	22.22	113.6	435.4	2.86	0.94	0.394	13.52	659.9	8.71
LP13	108.5	2353	5.33	23.69	137.2	588.9	3.04	1.25	0.581	17.63	594.9	9.41
LP14	131.3	2446	6.14	41.77	86.3	663.4	2.78	1.18	0.798	19.66	674.6	7.09*
LP15	130.4	2670	6.98	42.99	113.3	801.3	2.88	1.24	0.865	24.86	823.4	8.03
LP16	160.7	2618	2.96	30.97	166.1	263.5	5.19	2.26	0.276	12.45	1416.9	10.83
LP17	156.8	2696	10.08	38.83	93.0	675.7	8.80	2.88	0.485	13.84	2530.5	9.40
LP18	198.2	2623	2.93	62.10	443.5	667.7	5.07	1.32	0.353	22.71	1280.7	10.40
LP19	144.5	2051	6.77	23.25	103.1	829.6	4.28	1.93	0.784	14.04	841.5	7.50*
LP20	134.1	1863	3.18	29.01	94.5	417.4	2.97	2.18	0.296	28.67	740.7	7.72*
YV01	132.8	1591	2.42	18.47	72.3	252.2	3.25	0.94	0.169	11.03	687.6	7.04*
YV02	126.9	1372	3.22	18.92	103.0	251.2	3.35	1.02	0.186	10.92	672.9	7.16*
YV03	116.2	1792	2.39	19.47	61.9	295.6	2.93	1.00	0.260	12.93	629.8	7.23*
YV04	125.2	1677	2.44	18.15	87.2	300.6	3.67	0.82	0.174	8.59	628.0	7.23*
YV05	123.8	1764	3.74	13.49	94.9	461.9	2.30	1.22	0.231	7.52	483.0	6.74*
YV06	103.6	2166	4.45	27.10	64.2	446.9	4.21	1.12	0.381	16.35	1050.2	9.49

Wine	Cr	Mn	Co	Ni	Cu	Zn	As	Mo	Cd	Pb	Al	Se
YV07	99.7	1782	3.03	22.44	64.7	364.7	3.11	1.18	0.257	14.74	754.3	8.97
YV08	132.9	2440	3.16	21.28	80.0	336.2	5.51	1.24	0.241	16.93	886.9	8.07
YV09	116.6	2370	3.59	21.78	31.7	422.3	4.63	1.42	0.300	16.66	1148.4	7.15*
YV10	107.8	1500	2.57	18.83	28.3	333.3	4.37	1.35	0.167	7.86	648.5	6.79*
YV11	116.2	2274	2.11	24.11	59.1	217.7	5.13	1.36	0.252	16.58	638.6	9.53
YV12	107.5	1650	2.98	13.81	18.5	450.6	6.06	0.83	0.199	6.90	415.7	6.65*
YV13	132.9	2334	4.32	22.03	70.6	645.8	5.11	1.53	0.379	15.25	1091.1	7.90*
YV14	97.8	1558	2.40	14.90	67.0	264.6	4.01	2.02	0.238	17.53	697.2	10.30
YV15	145.1	1621	5.00	20.50	154.7	620.3	5.28	1.18	0.329	12.42	1094.2	7.60*
YV06	96.1	1802	4.32	16.10	114.4	493.1	6.58	1.39	0.291	9.50	468.7	9.31
YV17	147.8	1879	2.37	19.51	28.1	302.5	3.06	2.16	0.221	13.39	513.1	7.28*
YV18	159.1	1691	2.37	34.15	75.6	235.0	7.30	1.21	0.208	13.48	965.0	9.19
YV19	85.4	1619	2.91	21.94	100.9	294.0	3.22	1.02	0.192	11.11	660.8	9.15
YV20	133.0	1874	1.93	12.49	142.1	226.9	2.68	0.39	0.194	11.08	539.5	6.62*
YV21	85.2	2043	4.79	24.89	334.3	486.2	3.90	1.20	0.387	14.91	1209.6	9.04
YV22	100.8	1630	3.49	19.33	96.8	381.9	4.04	0.97	0.248	9.49	758.3	9.83
YV23	101.4	4771	6.03	56.44	78.2	671.2	3.80	1.20	1.025	25.17	746.7	9.91
YV24	111.1	1997	2.77	20.28	68.2	475.8	5.16	2.04	0.371	17.38	508.2	8.86
YV25	101.9	2492	4.00	34.51	84.5	413.7	4.85	1.46	0.434	18.41	886.4	9.76
YV26	91.1	2724	3.96	22.54	120.0	657.5	6.28	0.82	0.218	10.32	674.0	8.81
YV27	153.0	2319	2.90	21.71	192.8	357.0	2.83	0.91	0.305	16.02	501.1	9.78
YV28	146.9	2414	3.03	34.58	90.7	523.1	4.82	1.71	0.296	12.23	648.0	9.41
YV29	106.9	2809	2.84	17.47	174.3	420.2	2.88	0.71	0.281	15.35	581.8	9.60
YV30	162.3	2358	3.55	32.80	78.7	515.7	5.18	1.87	0.263	10.94	575.3	9.63
YV31	142.5	1261	3.57	18.46	70.7	658.6	4.56	1.75	0.375	16.54	825.2	9.55

Wine	Cr	Mn	Co	Ni	Cu	Zn	As	Mo	Cd	Pb	Al	Se
YV32	150.5	2517	3.85	23.58	139.4	536.1	5.29	1.27	0.224	12.48	853.6	10.17
YV33	143.6	2073	4.39	20.86	133.3	496.7	4.59	2.40	0.216	11.98	1131.8	9.97
YV34	155.5	2030	4.99	22.51	25.1	573.1	6.95	9.13	0.272	16.47	937.8	7.61*
YV35	146.1	1921	4.06	20.62	71.7	440.2	4.09	1.49	0.189	13.70	1012.8	8.06
BG01	137.6	5450	5.34	44.96	422.8	628.6	3.37	1.46	1.101	42.55	1206.2	8.45
BG02	134.9	3635	4.93	36.20	343.7	685.2	3.32	0.68	1.157	78.28	1104.8	8.48
BG03	121.7	2817	3.53	25.14	84.3	301.7	5.13	1.13	0.327	15.56	922.6	8.40
BG04	142.5	7679	9.80	93.64	107.0	794.6	4.28	1.22	1.555	25.36	1061.6	10.24
BG05	151.9	6957	11.35	131.67	89.1	860.3	6.56	1.89	1.918	33.60	1093.3	10.44
BG06	124.5	4404	4.20	36.36	89.5	402.9	9.31	1.24	0.992	22.27	905.2	7.63*
BG07	161.5	8436	11.06	106.82	92.6	973.7	6.45	1.11	2.356	27.80	1212.0	7.98*
BG08	158.3	8037	12.45	111.12	114.5	936.1	7.96	1.43	2.292	30.07	1322.5	8.39
BG09	118.8	3630	4.40	40.90	103.0	411.0	2.47	1.06	0.700	15.42	749.6	7.59*
BG10	115.6	6586	5.70	39.75	60.7	456.0	2.99	0.72	0.858	11.33	654.2	7.88*
BG11	192.6	9782	6.77	81.12	61.6	484.6	5.38	1.36	1.258	41.49	1386.6	10.40
BG12	189.2	12745	5.28	93.87	66.9	390.1	4.02	0.97	1.600	32.03	892.4	10.68
BG13	163.7	9418	9.37	162.00	77.8	744.0	4.83	1.50	1.562	29.40	1080.1	9.31
BG14	157.2	8546	8.54	71.93	83.2	501.5	3.65	0.97	1.057	32.96	928.8	10.60
BG15	146.4	7346	5.26	124.17	102.3	579.7	2.49	1.11	1.539	38.66	789.4	8.20
BG16	166.5	12460	8.31	96.60	32.7	713.9	6.03	2.02	2.039	44.76	1187.8	8.57
BG17	129.7	8399	5.52	52.05	51.3	415.6	2.13	0.76	0.902	15.07	639.0	7.75*
BG18	123.7	8893	5.82	56.00	36.4	759.4	2.16	0.50	1.289	18.05	645.3	7.67*
BG19	158.9	9856	2.94	45.96	45.7	157.9	2.90	0.72	0.649	19.61	793.8	9.61
BG20	119.0	2790	3.21	68.93	104.2	870.4	3.72	0.86	0.174	8.15	409.0	6.88*
BG21	128.1	3157	2.99	61.11	105.1	865.1	4.54	0.63	0.309	5.91	366.4	7.23*

Wine	Cr	Mn	Co	Ni	Cu	Zn	As	Mo	Cd	Pb	Al	Se
BG22	149.8	4867	4.40	45.80	58.6	881.2	8.44	2.34	0.708	31.96	766.0	8.30
BG23	149.2	6649	5.85	84.44	49.2	448.4	6.80	2.20	1.306	18.58	996.9	8.05
BG24	145.5	4657	5.04	53.81	38.7	999.0	8.89	2.44	0.777	30.04	780.6	7.70*
BG25	125.1	2431	3.73	55.40	113.0	551.6	25.76	2.35	0.718	24.06	884.2	9.12
BG26	166.2	5602	4.51	146.53	79.4	419.5	8.68	1.47	1.240	17.00	1487.9	10.50
BG27	129.2	2659	3.79	52.85	122.1	483.2	20.47	1.77	0.796	24.49	935.0	8.84
BG28	163.4	5620	4.69	72.65	89.4	713.6	31.40	1.21	1.443	21.21	1509.8	10.53
BG29	168.0	6426	4.26	82.55	56.4	362.3	7.37	1.01	0.880	20.35	818.7	10.23
BG30	160.4	9219	7.32	105.82	71.2	575.4	4.30	1.04	1.530	30.21	1116.5	10.06
BG31	169.0	8852	8.53	113.16	65.0	793.0	6.62	1.67	1.852	41.68	1271.6	10.32
BG32	156.9	8040	5.36	70.60	112.4	447.4	4.54	1.15	1.254	38.76	993.0	10.52
BG33	155.4	8291	6.11	49.37	225.1	414.4	3.17	1.09	0.805	15.34	765.3	9.95
BG34	161.8	5341	5.10	54.71	58.5	478.7	6.15	2.60	0.936	22.84	1461.8	10.35
BG35	172.0	6309	5.73	295.05	93.1	534.5	3.13	2.62	0.829	21.85	858.1	9.81
BG36	150.0	2904	3.61	33.98	114.7	490.4	2.71	0.69	0.465	13.84	656.7	10.57
BG37	144.0	11073	8.76	46.60	58.6	687.4	5.52	0.83	1.501	17.63	810.7	9.41
BG38	130.1	2772	6.25	39.61	135.1	540.3	4.49	1.87	0.857	31.48	917.9	7.96*
BG39	148.4	1046	3.27	22.04	77.9	515.5	5.40	1.39	0.514	24.07	1378.1	8.13
BG40	181.5	1591	3.03	32.08	113.9	427.4	3.04	0.79	0.486	24.28	1390.5	10.03
SWH01	148.8	2457	2.38	17.00	170.1	207.7	5.49	0.48	1.008	18.23	424.6	8.09
SWH02	94.5	1554	2.90	57.73	121.6	536.7	2.38	1.10	1.330	22.57	319.8	9.01
SWH03	207.0	3077	4.68	43.66	131.3	412.4	12.61	0.65	1.506	30.77	1008.5	6.89*
SWH04	135.1	2393	2.67	14.25	13.7	208.8	7.24	0.39	1.765	28.42	659.4	6.53*
SWH05	95.8	1264	2.46	22.06	72.4	230.2	12.50	0.97	2.162	26.15	2686.8	9.14
SWH06	96.6	2895	4.49	25.63	89.1	826.1	3.44	0.90	0.512	18.21	617.8	9.95

Wine	Cr	Mn	Co	Ni	Cu	Zn	As	Mo	Cd	Pb	Al	Se
SWH07	128.6	1599	2.73	21.27	58.2	272.2	3.47	0.39	0.816	14.81	481.6	6.83*
SWH08	141.4	2355	3.04	20.95	41.2	305.7	4.46	0.47	0.997	17.72	629.1	6.29*
SWH09	127.1	1990	2.74	28.54	94.0	382.4	6.54	1.00	0.758	15.16	539.3	9.28
SWH10	100.0	1404	2.37	17.66	285.3	198.9	3.98	0.76	0.265	15.34	566.8	9.63
SWH11	127.8	2097	2.93	21.69	91.3	332.3	5.46	0.83	0.660	12.63	464.8	9.16
SWH12	95.9	1568	2.55	21.50	92.5	322.1	5.27	1.28	1.622	11.15	440.3	6.06*
SWH13	106.4	2037	3.00	36.71	34.1	136.5	5.09	1.38	0.466	16.76	1209.9	7.95*
SWH14	102.6	1641	2.20	20.21	121.9	261.5	3.36	1.61	0.676	11.36	634.8	7.99*
SWH15	81.4	1740	4.27	24.90	137.8	276.7	4.90	0.66	0.453	10.13	389.1	8.62
SWH16	118.7	2538	5.07	36.54	35.2	773.7	6.98	1.38	0.970	20.52	1130.1	8.25
SWH17	146.4	1505	1.27	21.82	121.0	111.0	3.66	1.22	0.150	8.46	306.2	8.01
SWH18	89.9	1950	2.88	21.27	67.1	318.1	4.49	1.41	3.037	24.56	2972.8	8.99
SWH19	126.2	1185	2.24	33.27	72.4	660.7	10.81	6.14	1.071	27.27	1544.3	8.50
SWH20	95.5	2066	2.40	53.08	70.8	283.7	2.96	1.42	0.809	26.99	339.7	9.33
SWH21	95.4	2635	3.43	41.84	79.4	585.3	2.11	1.48	0.474	14.15	485.7	9.11
SWH22	94.0	2154	2.43	18.87	82.0	235.6	8.34	2.35	1.294	23.61	717.8	8.04
SWH23	96.7	1807	5.17	24.30	199.1	313.1	6.48	1.20	0.326	16.25	1118.9	9.72
SWH24	91.7	2032	2.41	17.86	71.1	308.2	5.37	0.99	1.030	22.47	4161.3	8.82
SWH25	123.2	2438	3.03	20.09	24.5	215.1	6.59	1.53	1.692	28.32	1839.6	7.23*
SWH26	454.1	1669	6.27	193.59	88.4	608.6	2.20	1.20	0.174	6.02	152.0	8.53
SWH27	159.9	2141	8.06	53.79	39.9	1981.4	4.23	1.23	0.348	19.91	700.3	7.69*
SWH28	190.2	2069	11.93	38.62	88.1	466.3	3.57	1.91	0.272	11.37	401.5	7.71*
SWH29	121.2	1777	2.88	18.39	37.4	621.5	5.18	2.85	0.380	7.52	273.7	6.90*
SWH30	132.8	2301	2.44	17.44	156.6	1235.0	5.89	3.40	0.335	9.06	394.7	7.27*
SWH31	150.0	2146	2.19	11.57	156.3	571.3	2.71	3.38	0.201	12.88	351.3	8.50

Wine	Cr	Mn	Co	Ni	Cu	Zn	As	Mo	Cd	Pb	Al	Se
SWH32	175.0	1838	2.46	26.07	78.9	430.7	6.22	2.90	0.200	13.09	550.7	8.93
SWH33	91.7	2341	2.87	19.45	138.6	393.0	6.00	2.32	0.229	10.65	608.3	9.18
SWH34	140.0	1755	3.51	13.28	238.9	492.7	2.12	0.55	0.207	19.65	324.8	6.07*
SWH35	134.6	2423	1.15	13.85	55.8	245.2	1.69	0.76	0.141	2.05	256.8	6.31*
SWH36	82.0	1721	2.19	17.54	131.3	422.1	2.28	0.99	0.161	5.52	143.6	8.49
SWH37	79.3	4649	2.64	22.66	211.6	574.8	1.88	0.86	0.452	6.59	378.6	7.90*
SWH38	85.2	1922	2.36	17.18	197.3	426.8	3.05	1.61	0.277	7.44	199.0	9.06
SWH39	77.4	1891	1.69	18.32	89.8	167.8	1.70	0.83	0.442	7.19	126.5	8.50
SWH40	76.0	1273	1.80	18.19	104.1	208.0	1.90	0.83	0.613	8.59	89.6	8.21
SWH41	89.6	1959	3.80	34.73	81.3	297.9	8.28	2.15	0.238	12.06	1359.4	12.75
SWH42	85.8	2267	2.10	21.94	140.9	577.4	2.15	1.06	0.758	10.30	328.1	8.74
SWH43	75.5	4016	2.91	20.74	138.8	929.9	2.69	1.04	1.535	20.80	353.1	8.57
SWH44	78.3	2627	2.35	21.10	165.0	545.9	2.17	1.17	0.761	10.98	283.2	8.49
SWH45	135.1	4780	3.01	27.12	148.3	921.1	2.94	1.19	1.392	20.79	441.3	9.16
SWH46	128.7	3029	5.95	29.79	251.7	476.0	2.08	0.79	1.351	14.41	312.4	9.83
SWH47	126.8	2312	1.83	14.83	150.9	417.7	2.37	1.68	0.196	5.80	245.8	9.85
SWH48	143.0	2789	2.16	16.41	176.7	546.3	1.91	1.69	0.155	3.21	216.6	7.93*
SWH49	164.5	3067	2.72	18.72	24.1	958.3	2.39	1.14	0.123*	3.76	278.3	8.79
SWH50	125.2	3257	2.28	10.38	120.8	590.5	2.23	1.36	0.100*	3.20	317.4	7.40*

Notes: The results of wines from different regions were the mean values in triplicate (HM: Helan Mountain; XJ: Xinjiang; HC: Hexi Corridor; BG: Bohai Gulf; SWH: Southwest Highland; YV: Yanhuai Valley; NE: Northeast; LP: Loess Plateau).

* The value was below to LOQ but higher than LOD as the estimated result.
